# Systemic inflammatory response syndrome in Sepsis-3: a retrospective study

**DOI:** 10.1186/s12879-019-3790-0

**Published:** 2019-02-11

**Authors:** Wei Zhang, Yan Zheng, Xiaoting Feng, Miao Chen, Yan Kang

**Affiliations:** 1 Department of Critical Care Medicine, Affiliated Hospital of Zunyi Medical College, Zunyi Medical College, 149 Dalian Road, Zunyi, 563000 Guizhou China; 2 Department of Ultrasound, Linyi City People’s Hospital, Linyi, 276000 Shandong China; 3 Department of Critical Care Medicine, Sichuan University West China Hospital, Chengdu, 610041 Sichuan China

**Keywords:** Sepsis, Intensive care unit, Critically ill patients, Systematic inflammatory response syndrome, Sequential (sepsis-related) organ failure assessment

## Abstract

**Background:**

In the new Sepsis-3 definition, sepsis is defined as “life-threatening organ dysfunction due to a dysregulated host response to infection.” We tested the predictive validity of the systematic inflammatory response syndrome (SIRS) criteria in patients in the Sepsis-3 cohort.

**Methods:**

Among 1243 electronic health records from 1 January to 31 December 2015 at Sichuan University West China Hospital, we identified patients with sepsis and septic shock according to the Sepsis-3 definition and divided them into 2 subsets: SIRS-positive and SIRS-negative. We compared their characteristics and outcomes as well as the predictive validity of the SIRS criteria for in-hospital mortality.

**Results:**

Of the 1243 patients, 631 were enrolled. Among these, 538 (85.3%) patients had SIRS-positive sepsis or septic shock, 168 (31.2%) of whom died, and 93 (14.7%) had SIRS-negative sepsis or septic shock, 20 (21.5%) of whom died (*p* = 0.06). Over a 1-year period, these groups had similar characteristics and changes in mortality. Among patients of the Sepsis-3 cohort admitted to the intensive care unit, the predictive validity for in-hospital mortality was lower for the SIRS criteria (area under the receiver operating characteristic curve [AUROC], 0.53; 95% confidence interval [95% CI], 0.49–0.57) than for the sequential (sepsis-related) organ failure assessment (SOFA) criteria (AUROC, 0.70; 95% CI, 0.66–0.74; *p* ≤ 0.01 for both). The SIRS score had poor predictive validity for the risk of in-hospital mortality.

**Conclusions:**

In this cohort study of the new Sepsis-3 definition, we found that the SIRS criteria are weaker than the SOFA criteria with respect to their predictive efficacy for in-hospital death.

## Background

Despite considerable medical advances, sepsis is common and associated with high morbidity and mortality rates [1, 2]. In 1991, the Task Force in the First International Consensus Conference used expert opinion to generate the then-current definitions of sepsis (First International Consensus Conference Definitions for Sepsis and Septic Shock [Sepsis-1]) based on the presence of systematic inflammatory response syndrome (SIRS) [3]. Because of high sensitivity and low specificity, Sepsis-1 was replaced by Sepsis-2 in 2001 [4]. However, Sepsis-2 did not show superiority over Sepsis-1 in the diagnosis of sepsis [5]. In 2015, a study of SIRS in patients with severe sepsis completely disclosed the flaws of the SIRS criteria, prompting further revision of the sepsis definition [6]. In 2016, the Third International Consensus Conference established a new sepsis definition (Sepsis-3) [7]. In Sepsis-3, the Sequential (Sepsis-related) Organ Failure Assessment (SOFA) criteria, rather than the SIRS criteria, are used as the basis for the definitions of sepsis and septic shock.

Since application of the SIRS criteria to the definition of sepsis during the past two decades, many clinicians have become ingrained in thinking that the pathophysiological condition progresses from SIRS to sepsis and septic shock and then to multiple organ failure [8–10]. However, sepsis is actually a syndrome of severe infection with a complicated pathogenesis beyond the scope of our recognition [11]. Many experts and specialists have attempted to use the clinical criteria of SIRS to describe the pathophysiological process and nature of inflammatory syndromes caused by severe infection, but the outcomes have been unsatisfactory [5]. A new definition of sepsis derived from a database of developed countries has been validated for use in these developed countries [12]. However, it is necessary to be further validated for the concept whether can be generalized to developing countries. Certainly, some studies evaluated SEPSIS-3 in developing countries have aroused our attention [13, 14]. In the present study, we used data from developing countries to compare the SIRS criteria with the SOFA criteria to predict a high risk of in-hospital death among critically ill patients with sepsis according to the new definition.

## Methods

### Study design and setting

This retrospective study was conducted in a general intensive care unit (ICU) and included adult patients with sepsis or septic shock according to the Sepsis-3 definition from 1 January to 31 December 2015, using data from the Sichuan University West China Hospital Critical Care Medicine Sepsis-3 Database. This study was approved by the Ethics Committee of Sichuan University West China Hospital (No. 315, 2016). Due to the retrospective study design involving electronic health records and no additional interventions, written informed consent was not obtained from the patients or their relatives. This study was registered at the Chinese Clinical Trial Register (CCTR number: ChiCTR-ORC-16010138, registered 12 December 2016). URL: http://www.chictr.org.cn/showproj.aspx?proj=16715.

### Participants

#### Inclusion criteria

The inclusion criteria of the study were as follows:

1. Age of ≥18 years
2. A ≥ 24-h stay in the general ICU
3. The presence of infection or suspected infection, defined as follows [12]: 1) The initial episode of suspected infection was identified through a combination of antibiotic treatment and body fluid cultures. 2) We required that the combination of antibiotics and culture sampling occurred within a specific time limit. If the culture sampling occurred first, antibiotic must have been administered within 72 h. If the antibiotic was administered first, the culture sampling must have been obtained within 24 h. 3) The onset of infection was defined as the time point at which the first of the two events (antibiotic treatment and culture sampling) took place.

### Primary and secondary outcomes

In this study, we regarded SIRS-positive sepsis as the primary outcome and followed up all patients before hospital discharge using their medical records. All-cause in-hospital mortality was the secondary outcome.

### Definition of cohorts

Indicators were generated for each component of the SIRS criteria [6] and SOFA score [15]. We calculated the maximum SIRS criteria and SOFA score for the time window ranging from 48 h before to 24 h after the onset of infection. Organ dysfunction in patients with sepsis occurring before, near the moment of, or after infection is recognized by clinicians. Thus, for the candidate criteria, we used that time window. From up to 48 h before to up to 24 h after the onset of infection, we calculated changes of ≥2 points in the SOFA score [7, 12].

We defined sepsis or septic shock according to the Sepsis-3 definitions [7]. Organ dysfunction can be identified as an acute change of ≥2 points in the total SOFA score consequent to the infection. The baseline SOFA score can be assumed to be 0 points in patients not known to have pre-existing organ dysfunction. Even patients presenting with modest dysfunction can deteriorate further, emphasizing the seriousness of this condition and the need for prompt and appropriate intervention if not already being instituted. Septic shock is a subset of sepsis in which underlying circulatory and cellular/metabolic abnormalities are profound enough to substantially increase mortality. Patients with septic shock can be identified using a clinical construct of sepsis with persisting hypotension requiring vasopressors to maintain the mean arterial pressure at ≥65 mmHg and serum lactate concentration at > 2 mmol/L (18 mg/dL) despite adequate volume resuscitation.

Among the Sepsis-3 cohort, the SIRS-positive cohort was defined as patients with SIRS scores of ≥2 points, and the SIRS-negative cohort was defined as patients with SIRS scores of < 2 points, including those with scores of 0 points and 1 point [6].

### Data collection

We collected general information including medical identification numbers, demographic characteristics, vital signs, and laboratory test results from the medical records of patients upon admission to the ICU or during their stay in the ICU. We calculated the SIRS and SOFA scores for each patient using these data. Acute Physiology and Chronic Health Evaluation II (APACHE II) [16] scores were collected to assess the severity of illness among patients admitted to the ICU in the first 24 h.

### Bias

Researchers who participated in data collection for the study were blinded to the study design, and the study designers did not participate in the data collection.

### Statistical analysis

Data are presented as number and percentage, mean and standard deviation, median and interquartile range, or proportion with 95% confidence interval. The chi-square test for equal proportion, Student’s t-test, or the Wilcoxon rank-sum test was used to test differences. No assumptions were made for missing data, and multivariable analyses were performed for patients with complete data.

To identify independent differences at baseline that may have existed between patients with SIRS-positive sepsis and SIRS-negative sepsis, we applied multivariable logistic regression to the data from all patients with severe sepsis with a SIRS-positive status as the outcome. To further determine the predictive capacity of using two or more SIRS criteria to identify an increase in the risk of death, SIRS was considered first as a dichotomous variable (≥2 SIRS criteria vs. 0 to 1 SIRS criterion) and second as an ordinal variable from 0 to 4, reflecting the number of SIRS criteria met. To determine whether predictors of death differed significantly between SIRS-positive sepsis and SIRS-negative sepsis, we created a multivariable logistic regression model for mortality among all patients with sepsis. All statistical analyses were performed using MedCalc® (version 15.8) statistical software [17] and Empower Stats software [18]. All statistical tests were two-tailed, and *p* < 0.05 was considered significant.

## Results

### Study cohort characteristics

**Fig. 1:**
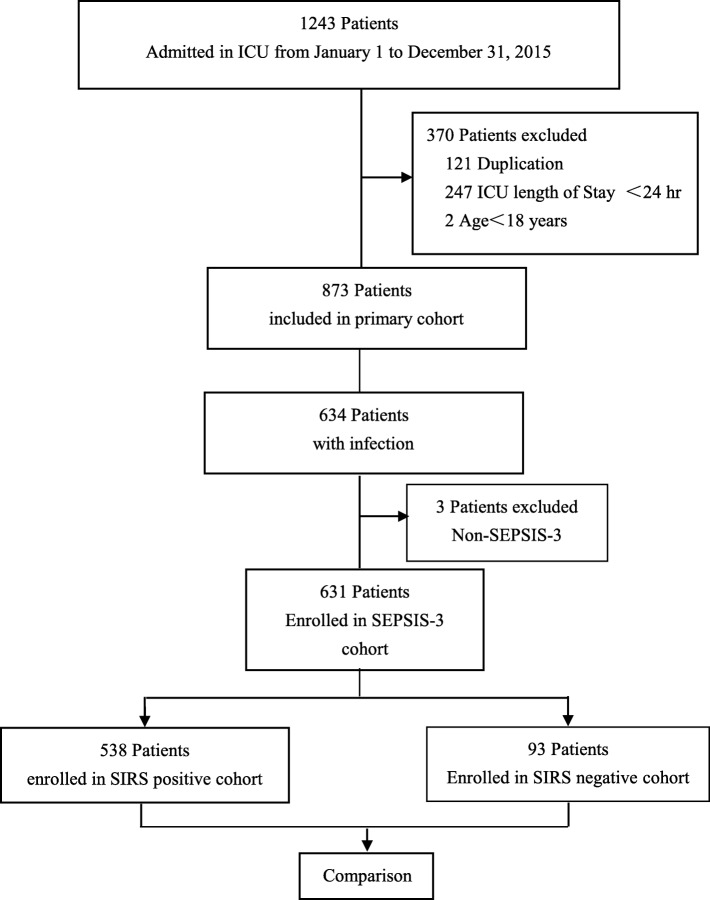
Flow diagram of the study

A flow diagram of the study is shown in Fig. 1. As described in the Methods section, 1243 patients were evaluated in the enrollment period and 873 patients had complete clinical data. We finally enrolled 631 patients with sepsis or septic shock according to the Sepsis-3 definition. In total, 370 patients were excluded because of a < 24-h ICU length of stay (*n* = 247), secondary admission to the ICU (*n* = 121), and an age of < 18 years (n = 2). Of 538 patients enrolled in the.

SIRS-positive cohort, 168 (31.2%) died, and of 93 patients enrolled in the SIRS-negative cohort, 20 (21.5%) died; the outcome of pairwise assessment revealed no significant difference (*p* = 0.06). There were no missing data.

### Baseline risk

The patients’ baseline characteristics are listed in Table 1. The patients’ age was higher in the SIRS-negative cohort than in the SIRS-positive cohort (*p* = 0.011). The SIRS scores were higher in the positive than negative cohort. The median APACHE II score for all patients upon ICU arrival was 25. SOFA scores were available for 631 patients, and the median was 9. The SOFA and APACHE II scores were not significantly different between the two cohorts. The median length of ICU stay was 13 days (range, 7–24 days); it was also 13 days in the SIRS-positive and -negative cohorts separately, and pairwise comparison showed no significant difference (*p* = 0.622). The median length of hospital stay was 22 days (range, 12.5–35 days); it was 22 and 20 days in the SIRS-positive and -negative cohorts, respectively, and pairwise comparison showed no significant difference (*p* = 0.569). The proportion of male patients was 66.1% (417 of 631), and there was no significant difference in the proportion of male patients between the SIRS-positive and -negative cohorts (*p* = 0.412). The median 28-day of ventilator-free days and the duration of continuous renal replacement therapy were 8 and 10 days, respectively, with no significant differences between the two SIRS cohorts. The rates of mechanical ventilation and continuous renal replacement therapy in patients with sepsis were not significantly different between the cohorts (both *p* > 0.05). However, the rate of septic shock in patients with sepsis was higher in the SIRS-positive than SIRS-negative cohort (*p* = 0.044).

**Table 1:** Baseline characteristics and hospital outcomes of patients with sepsis^a^

Characteristic	All Patients		Patients with SIRS-Positive Sepsis		Patients with SIRS-Negative Sepsis		*P* Value
	No. of patients with data		No. of patients with data		No. of patients with data		
Age-yr	631		538		93		0.011
Median		60.0		60		64	
Interquartile range		46.0–73.0		45.0–73.0		50–77.0	
Male sex — no. (%)	631	417 (66.1%)	528	359 (66.7%)	92	58 (62.4%)	0.412
Risk of death-%							
APACHE II	631		538		93		0.087
Median		25.0		25		24	
Interquartile range		20.0–29.0		20.-29.		20.-28	
SOFA	631		538		93		0.05
Median		9		9		9	
Interquartile range		7–12		7–13		7–11	
SIRS	631		538		93		< 0.001
Median		3.0		3.0		1.0	
Interquartile range		2.0–3.0		2.0–3.0		1.0–1.0	
Duration of stay							
In ICU-days	631		538		93		0.622
Median		13.0		13.0		13.0	
Interquartile range		7.0–24.0		7.0–24.0		8.0–22	
In-hospital-days	631		538		93		0.569
Median		22.0		22.0		20.0	
Interquartile range		12.5–35.0		12.0–36		13.0–28.0	
28-day of ventilator-free days	573		489		84		0.159
Median		8.0		9.0		8.0	
Interquartile range		4.0–16.0		4.0–17.0		3.0–14.0	
Duration of CRRT-days	109		96		13		0.560
Median		10		8.5		14.0	
Interquartile range		5.0–20.0		5.0–17.0		10.0–23.0	
Hospital outcome	631		538		93		0.058
Death-%		188 (29.8)		168 (31.2)		20 (21.5)	
Subgroup- no(%)	631		538		93		
Septic shock		212 (33.6)		189 (35.1)		23 (24.7)	0.044
Acute kidney failure of CRRT		109 (17.3)		96 (17.8%)		13 (14.0%)	0.363
Mechanical ventilation		573(90.8)		489(90.9)		84(90.3)	0.861

^a^ Plus–minus values are means ±SD. SIRS-positive status was defined if the patient fulfilled at least two SIRS criteria, and SIRS-negative status if the patient fulfilled zero or one SIRS criterion. ICU denotes intensive care unit

Scores on the APACHE II range from 0 to 71, with higher scores indicating more severe disease

Abbreviation: *APACHE* Acute Physiology and Chronic Health Evaluation, *SOFA* Sepsis-related Organ Failure Assessment, *SIRS* Systemic Inflammatory Response Syndrome, *CRRT* Continuous renal replacement therapy. Normal distributed data are expressed as mean ± standard deviation

### Distribution of hospital mortality

**Fig. 2:**
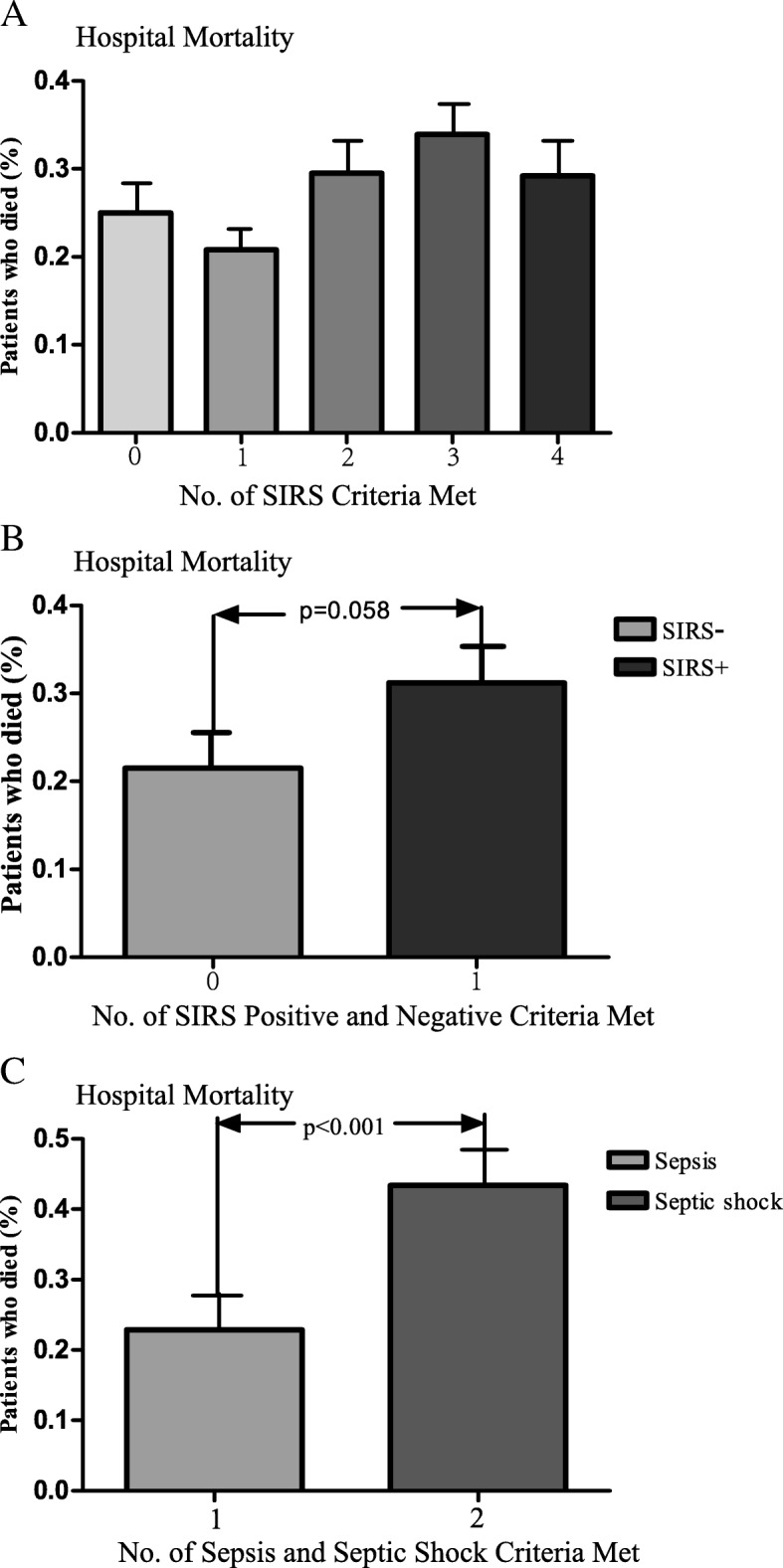
Distribution of hospital mortality

**Fig. 3:**
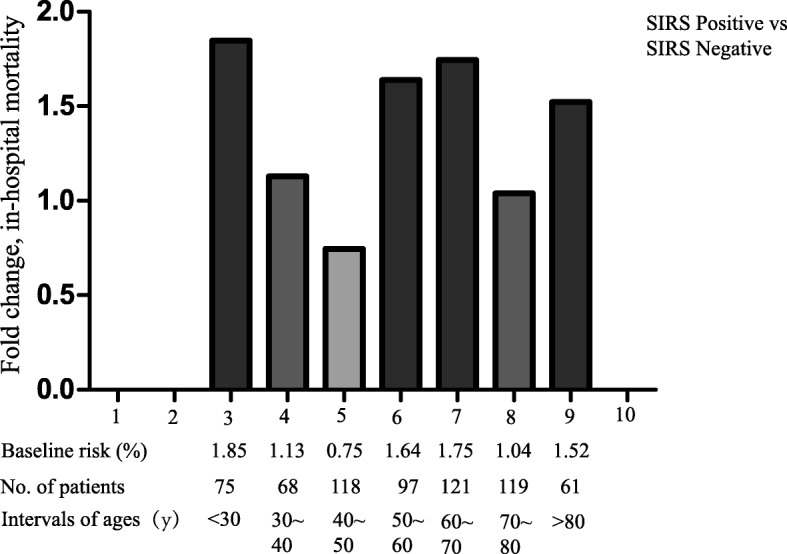
Fold change (ratio) hospital mortality of patients with SIRS positive (scores ≥2) vs. SIRS negative (< 2)

The distributions of hospital mortality according to the SIRS score and subsets of the new Sepsis-3 definition are shown in Fig. 2. An increasing trend of hospital mortality with increasing SIRS scores was not evident (*p* > 0.05) (Fig. 2a). This held true for both the SIRS-positive and -negative cohorts (Fig. 2b). However, the distribution of hospital mortality was higher in the subgroups of patients with septic shock than in the subgroups of patients with sepsis (*p* < 0.001) (Fig. 2c). Among all age interval subgroups, the fold changes (ratio) of hospital mortality (SIRS score of ≥2 vs. < 2) were higher in the intervals of 3, 6, and 7 than in the other intervals (Fig. 3).

### SIRS in new Sepsis-3 definition

The distributions of signs meeting the SIRS criteria are shown in Table 2. The most frequent SIRS criterion that was met in patients with SIRS-positive sepsis was an increased heart rate, followed by an increased respiratory rate or a low partial pressure of arterial carbon dioxide and an abnormal white cell count. As in the patients with SIRS-positive sepsis, the most frequent single criterion that was met in patients with SIRS-negative sepsis was an increased heart rate (Table 2). Of patients with SIRS-negative sepsis, 17.2% fulfilled no SIRS criteria, and 82.8% fulfilled one SIRS criterion (Table 2).

**Table 2:** Distribution of signs meeting SIRS criteria in patients with sepsis or septic shock*

Variable	All Patients (n = 631)	Patients with SIRS-Positive Severe Sepsis (*n* = 538)	Patients with SIRS-Negative Severe Sepsis (*n* = 93)
SIRS criterion met — no. (%)^a^			
Abnormal temperature	630 (99.8)	250 (46.5)	6 (6.5)
Increased heart rate	631 (100)	506 (94.1)	43 (46.2)
Increased respiratory rate or decreased PaCO_2_	630 (99.8)	448 (83.3)	15 (16.1)
Abnormal white-cell count	629 (99.7)	331 (61.6)	13 (14.1)
No. of SIRS criteria met			
Median	3	3	1
Interquartile range	2–3	2–3	1–1
Distribution			
> 1	538 (85.3)	538 (85.3)	0
0	16 (2.5)		16 (17.2)
1	77 (12.2)		77 (82.8)
2	200 (31.7)	200 (37.2)	0
3	218 (34.5)	218 (40.5)	0
4	120 (19.0)	120 (22.3)	0

* *P* < 0.001 for all comparisons between the SIRS-positive group and the SIRS-negative group. PaCO_2_ denotes partial pressure of arterial carbon dioxide

^a^ SIRS criteria are defined in the Supplementary Appendix. Patients may have more than one criterion

### Multivariate logistic regression analysis

The outcomes of the multivariate logistic regression analysis of hospital mortality and SIRS positivity are shown in Table 3. The risk factors for hospital mortality, including the APACHE II score, length of hospital stay, length of ICU stay, 28-day mechanical ventilation, rate of mechanical ventilation, administration of vasopressors, and SOFA score, were statistically significant (*p* < 0.05). The risk factors for SIRS positivity, including the SOFA score and hospital length of stay, were also statistically significant (*p* < 0.05).

**Table 3:** Outcomes of multivariate logistic regression analysis for the risk factors on hospital mortality and SIRS positive

Variables	Hospital mortality		SIRS positive	
	Odds ratio(95% CI)	*P* value	Odds ratio(95% CI)	*P* value
Age	1.00 (0.97, 1.02)	0.97	0.98 (0.94, 1.02)	0.32
Sex	1.04 (0.41, 2.63)	0.94	1.40 (0.35, 5.61)	0.63
APACHE II	1.09 (1.02, 1.16)	0.01	1.02 (0.92, 1.12)	0.75
qSOFA	1.18 (0.90, 1.55)	0.23	2.92 (2.06, 4.13)	<0.01
Hospital length of stay	0.96 (0.94, 0.98)	<0.01	0.97 (0.94, 0.99)	0.01
ICU length of stay	1.03 (1.00, 1.05)	0.02	1.00 (0.99, 1.01)	0.93
28-day of ventilator-free days	1.01 (0.95, 1.07)	<0.01	1.02 (0.99, 1.05)	0.16
Mechanical ventilation	3.33 (1.15, 9.64)	0.03	0.60 (0.26, 1.36)	0.22
Duration of CRRT	0.98 (0.94, 1.01)	0.20	1.02 (0.97, 1.06)	0.45
**CRRT	1.88 (0.28, 12.60)	0.51	1.53 (0.12, 19.72)	0.74
SOFA	1.18 (1.12, 1.25)	<0.01	0.98 (0.90, 1.06)	0.60
Vasopressors	2.67 (1.87, 3.80)	<0.01	1.34 (0.71, 2.55)	0.37
SIRS	1.06 (0.87, 1.29)	0.56	–	–

Abbreviation: *APACHE* Acute Physiology and Chronic Health Evaluation, *SOFA* Sepsis-related Organ Failure Assessment, *SIRS* Systemic Inflammatory Response Syndrome, *qSOFA* quick Sepsis-related Organ Failure Assessment, *CRRT* Continuous renal replacement treatment. Normal distributed data are expressed as mean ± standard deviation

### Predictive efficacy

**Fig. 4:**
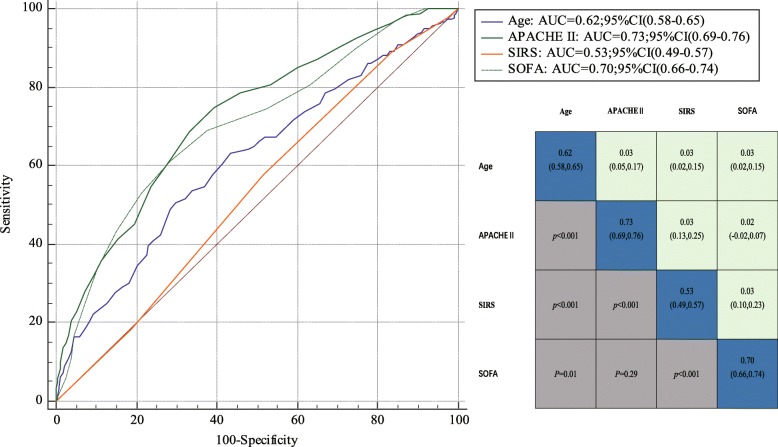
Area under the receiver operating characteristic curve 95% confidence intervals for hospital mortality of candidate criteria (Age, APACHE II, SOFA, and SIRS) among patients with Sepsis-3 cohort (*N* = 631).

The areas under the receiver operating characteristic curves (AUROCs) for the baseline risk model (age for mortality), SIRS, SOFA score, and APACHE II score are shown in Fig. 4. The AUROC for the SIRS model was 0.53 for prediction of hospital mortality; this was much lower than those for the baseline risk model, APACHE II score, and SOFA score (SIRS vs. age: 0.53 vs. 0.62, *p* < 0.01; SIRS vs. APACHE II: 0.53 vs. 0.73, *p* < 0.01; SIRS vs. SOFA: 0.53 vs. 0.70, *p* < 0.01).

## Discussion

In the present study, we found that the SIRS criteria had poor predictive validity for hospital mortality in critically ill patients with sepsis compared with the SOFA criteria under the new Sepsis-3 definition.

Among ICU patients with sepsis according to the new Sepsis-3 definition, 85.3% had SIRS-positive sepsis or septic shock and 14.7% had SIRS-negative sepsis or septic shock. Using the SIRS assessment system as a screening tool, we may miss approximately one in six of patients with infections at a high risk of death; this suggests that the SIRS assessment system may be unfit for critically ill patients with infections in ICUs of developing countries. In 2015, Kaukonen et al. (6) reported that the use of two or more SIRS criteria to define severe sepsis excluded one in eight otherwise similar patients with infection, organ failure, and substantial mortality. In the present study, we found that by using the SIRS system, we may miss more than one in six patients with infections at high risk of death. The rate of exclusion of those patients with a high risk of death was higher than reported by Kaukonen et al. (6) because we included the subset of patients with septic shock rather than severe sepsis of the Sepsis-2 definition.

In the present investigation, the AUROC for SIRS was much lower than in the Kaukonen’s study of predicting hospital mortality in ICU patients with sepsis (6). One reason for the discrepancy may be that the severity of illness in the patients of the present study was greater than that in the study by Kaukonen et al. (6) (APACHE II scores of ≥24: 55.6% vs. 28.8%, respectively).

Discrimination of hospital mortality using SIRS was much lower than that using SOFA. With respect to discrimination of hospital mortality using SIRS, we found that the AUROC was much lower than that for SOFA.

### Strengths

Our study has several strengths. First, the study is recent, making the data current and relevant. Second, it investigated the effect of the SIRS criteria within the time window of the initial episode of suspected infection during the ICU stay on the diagnosis of sepsis over a period of 1 year. Third, and most importantly, the SIRS data consisted of physiological or laboratory measurements that were retrospectively collected for routine monitoring indicators and are therefore unlikely to be biased.

### Limitations

The first limitation of this study is that symptoms meeting the SIRS criteria were evaluated only during the episode of suspected infection in the ICU as recorded every 1 or 2 h on the observation charts. The second limitation is that we conducted a single-center clinical investigation in a province of southwest China; thus, the characteristics of our study population may lack representativeness. Multicenter prospective studies could address this issue. Finally, the third limitation is the high mortality rate, which had two main causes. First, as a large tertiary teaching hospital, our institution receives numerous patients with severe infections who have been transferred from smaller hospitals and primary healthcare institutions. Second, the limitations of the SOFA system in a retrospective cohort study played an important role in the high morbidity.

### Generalization

Despite the above-described limitations, we investigated the potential prognostic value of the SIRS criteria in a relatively high-risk population and found it to be different from the SOFA criteria.

## Conclusions

In this cohort study of the new Sepsis-3 definition, we found that the SIRS criteria are weaker than the SOFA criteria with respect to their predictive efficacy for in-hospital death.

## Abbreviations

APACHE: Acute physiology and chronic health evaluation score
AUROC: Areas under the receiver operating characteristic curve
CCTR: Chinese clinical trial register
ICU: Intensive care unit
SIRS: Systematic inflammatory response syndrome
SOFA: Sequential (sepsis-related) organ failure assessment
